# Analysis of Resin-Dentin Interface Morphology and Bond Strength Evaluation of Core Materials for One Stage Post-Endodontic Restorations

**DOI:** 10.1371/journal.pone.0086294

**Published:** 2014-02-28

**Authors:** Kerstin Bitter, Christin Gläser, Konrad Neumann, Uwe Blunck, Roland Frankenberger

**Affiliations:** 1 Department for Operative and Preventive Dentistry, University School of Dental Medicine, CharitéCentrum 3, Charité - Universitätsmedizin Berlin, Berlin, Germany; 2 Department of Medical Informatics Biometry and Epidemiology, CharitéCentrum 4, Charité - Universitätsmedizin Berlin, Berlin, Germany; 3 Department of Operative Dentistry and Endodontology, University of Marburg, Marburg, Germany; University Hospital of the Albert-Ludwigs-University Freiburg, Germany

## Abstract

**Purpose:**

Restoration of endodontically treated teeth using fiber posts in a one-stage procedure gains more popularity and aims to create a secondary monoblock. Data of detailed analyses of so called “post-and-core-systems” with respect to morphological characteristics of the resin-dentin interface in combination with bond strength measurements of fiber posts luted with these materials are scarce. The present study aimed to analyze four different post-and-core-systems with two different adhesive approaches (self-etch and etch-and-rinse).

**Materials and Methods:**

Human anterior teeth (n = 80) were endodontically treated and post space preparations and post placement were performed using the following systems: Rebilda Post/Rebilda DC/Futurabond DC (Voco) (RB), Luxapost/Luxacore Z/Luxabond Prebond and Luxabond A+B (DMG) (LC), X Post/Core X Flow/XP Bond and Self Cure Activator (Dentsply DeTrey) (CX), FRC Postec/MultiCore Flow/AdheSE DC (Ivoclar Vivadent) (MC). Adhesive systems and core materials of 10 specimens per group were labeled using fluorescent dyes and resin-dentin interfaces were analyzed using Confocal Laser Scanning Microscopy (CLSM). Bond strengths were evaluated using a push-out test. Data were analyzed using repeated measurement ANOVA and following post-hoc test.

**Results:**

CLSM analyses revealed significant differences between groups with respect to the factors hybrid layer thickness (p<0.0005) and number of resin tags (p = 0.02; ANOVA). Bond strength was significantly affected by core material (p = 0.001), location inside the root canal (p<0.0005) and incorporation of fluorescent dyes (p = 0.036; ANOVA). CX [7.7 (4.4) MPa] demonstrated significantly lower bond strength compared to LC [14.2 (8.7) MPa] and RB [13.3 (3.7) MPa] (p<0.05; Tukey HSD) but did not differ significantly from MC [11.5 (3.5) MPa].

**Conclusion:**

It can be concluded that bond strengths inside the root canal were not affected by the adhesive approach of the post-and-core-system. All systems demonstrated homogenous hybrid layer formation and penetration into the dentinal tubules in spite of the complicating conditions for adhesion inside the root canal.

## Introduction

Prospective clinical investigations of endodontically treated teeth restored with adhesively-luted fiber reinforced composite posts (FRC posts) revealed survival rates of approximately 90% between 3 to 7 years [1]–[3]. However, a recently published observational clinical study demonstrated an annual failure rate of 4.6% after ten years [4]. The most frequently occurring failure modes were post debonding and post fracture. Consequently, it can be concluded that bonding of posts to root canal dentin is still a challenge due to limited access, visibility, moisture control, reduced number of dentinal tubules in the apical third of the root, and deposition of cementum and secondary dentin [5]. In addition, the C-factor inside the root canal has been shown to be extremely high [6]. Therefore, the achievement of reliable bonding and effective adhesion inside the root canal is still an issue of interest [6], [7]. With the aim to analyze effective hybridization of several adhesive and luting systems to dentin confocal laser scanning microscopy (CLSM) has been used for the investigation of the distribution of primer, adhesive and resin cement inside the hybrid layer and the dentinal tubules [8]–[11].

An advantage for the clinical application of fiber posts would be to combine luting of the fiber post inside the root canal and performing the core build-up in a one-stage procedure. This would be a time-saving and facilitating approach. Consequently, various manufacturers provide post-and-core systems and recommend the above mentioned procedure. This combination has been described as a secondary monoblock [7]; however, a previous study pointed out possible negative effects of core materials for luting fiber posts due to the higher filler content [12].

Post-and-core-systems are available with different adhesive approaches, i. e. self-etching or etch-and-rinse approach. Evaluation of both adhesive approaches for bonding fiber posts inside the root canal revealed conflicting results with either no difference between the systems [13], higher performance of self-etching adhesives [14] or higher bond strength for the etch-and-rinse approach compared to the self-etching approach [15]. The appearance of inhomogeneities inside the cement layer of adhesively luted fiber posts has been described in the literature [16], [17]. However, the effect of the occurrence of voids inside the cement layer remains controversial.

The first aim of the present study was to analyze morphological characteristics of resin-dentin interfaces with respect to hybrid layer thickness and penetration ability of four different post-and-core systems using two adhesive approaches. The second aim was to investigate the bond strength of labeled and microscopically analyzed specimens as well as of unlabeled specimens to the root canal dentin. Moreover, the appearance of inhomogeneities should be analyzed. This approach should identify whether any correlations between morphological characteristics and bond strengths as well as possible effects of fluorescent dyes and CLSM analyses on bond strengths exist. The null hypothesis was that bond strengths to root canal dentin would be not influenced by material, location inside the root canal, incorporation of fluorescent dyes, and CLSM analyses itself.

## Materials and Methods

### Specimen preparation

Eighty sound human maxillary central incisors were obtained with written informed consent under an ethics-approved protocol (EA1/034/06) by the Ethical Review Committee of the Charité - Universitätsmedizin Berlin and stored in 0.5% chloramine solution for at most one year post extractionem. The crowns of the teeth were sectioned at the proximal cemento-enamel junction using a diamond blade under constant water cooling. Root canal preparation was performed at a working length of −1 mm from the apical foramen using MTwo and FlexMaster rotary instruments (VDW, Munich, Germany) up to size .02/50. Working length was established using a C-Pilot file ISO 10 (VDW) that was inserted into the root canal until it could be visualized at the apical foramen. The working length was determined by subtracting 1 mm from this length. The root canals were filled with warm, vertically condensed gutta-percha (BeeFill®2in1, VDW) and AH Plus sealer (Dentsply DeTrey, Konstanz, Germany), and stored in water for 24 h to guarantee complete setting of the sealer and to avoid drying-out of the teeth. The apical foramen was sealed using the adhesive system Optibond FL (Kerr, Orange, CA, USA) according to the manufacturer's instructions.

The specimens were randomly divided into four groups (G) of 20 teeth each: G RB: Rebilda Post/Rebilda DC/Futurabond DC (Voco, Cuxhaven, Germany), G LC: Luxapost/Luxacore Z/Luxabond Prebond and Luxabond A+B (DMG, Hamburg Germany), G CX: X Post/Core X Flow/XP Bond and Self Cure Activator (SCA) (Dentsply Detrey, Konstanz, Germany), G MC: FRC Postec/MultiCore Flow/AdheSE DC (Ivoclar Vivadent, Schaan, Liechtenstein) (Table 1).

**Table 1:** Composition of materials used according to manufacturers except reference is provided.

Adhesive (Lot#)	Core material/(Lot#)	Manufacturer	Composition of adhesive	Composition of core material	Adhesive approach	Filler content and Flexural strength Core material
Futurabond DC (0946262, 0946263)	Rebilda DC (0951232)	Voco, Cuxhaven, Germany	**Futurabond DC:** organic acids, BIS-GMA, HEMA, TMPTMA, BHT, ethanol, fluorides, CQ, amine, catalysts	**Rebilda DC:** BIS-GMA, UDMA, DDDMA, BHT, dibenzoylperoxide, CQ, silica, bariumborosilicate glass ceramic, accelerators	Self-etch	71 wt% **Flexural strength:** 102 MPa
AdheSE DC (L22510, L32948, L28890)	Multicore Flow (L37355)	Ivoclar Vivadent, Schaan, Liechtenstein	**AdheSE Primer:** phosphonic acid, acrylate, dimethacrylates, water, **AdheSE Bond:** Bis-acrylamide, initiators, stabilizer, , HEMA, silicon dioxide, initiators, stabilizer **AdheSE Activator:** initiators, solvents, ethanol	**Multicore DC:** dimethacrylate, barium glass, fillers, Ba-Al-fluorosilicate glass, silicon dioxide, ytterbium trifluoride, catalysts, stabilizer, pigments	Self-etch	Base: 54.9 wt% Catalyst: 54.4 wt% **Flexural strength:** 135 MPa
Pre-Bond (635704) Luxabond A+B (635704)	Luxacore Z (635729)	DMG, Hamburg, Germany	**Prebond:** Ethanol Arylsulfinate solution **Luxabond A:** hydrophile Bis-GMA based resin matrix, catalyst **Luxabond B :** hydrophile BIS-GMA based resin matrix, benzoyl peroxide	**Luxacore Z:** barium glass, pyrogenic silicic acid, nano fillers, zirconium oxide in a Bis-GMA based resin matrix	etch-and-rinse	71 wt% **Flexural strength:** 150 MPa
XP Bond (0811001247) Self Cure Activator (080624)	CoreX Flow (0809111)	Dentsply DeTrey, Konstanz, Germany	**XP Bond:** PENTA, TCB, HEMA, TEGDMA, UDMA, tert-butanol, nanofiller, CQ, stabilizer **Self Cure Activator:** HEMA, UDMA, Catalyst, aromatic sodium sulfinate, Photoinitiator, Stabilizers, Acetone, Water	**CoreXFlow:** UDMA, di- and tri-functional methacrylates, Barium Boron Fluoroaluminosilicate glass, CQ, photoaccelerators, silicon dioxide benzoylperoxide	etch-and-rinse	70 wt% **Flexural strength:** 120 MPa

The following abbreviations are used: BHT: butylhydroxytoluene, BIS-EMA: ethoxylated bisphenol A glycol dimethacrylate, Bis-GMA: bisphenol A diglycidyl methacrylate, CQ: camphorquinone (photoinitiator), DDDMA: dodecanediol dimethacrylate, HEMA: 2-hydroxyethyl methacrylate, PENTA: dipentaerythritol pentaacrylate monophophate, UDMA: urethane dimethacrylate, TCB: butan-1,2,3,4-tetracarboyxlic di-2-hydroxyethylmethacrylate ester, TEGDMA: triethylene glycol dimethacrylate, TMPTMA: trimethylolpropane trimethacrylate.

Guttapercha was removed and the root canals were enlarged solely with a slow-speed drill provided by the manufacturer of the selected post-and-core system. The depth of the post space preparation was 8 mm that has been shown to be a clinically relevant length for post space preparation [3]. Irrigation after post space preparation was performed using 5 mL 0.9% NaCl solution; the fiber posts were tried-in and inserted with one of the four investigated post-and-core systems and the corresponding adhesive systems according to the manufacturers' recommendations. The core materials were applied onto the posts' surfaces and into the orifice of the root canals using an applicator that was autoclaved after each use. The posts were inserted into the canal. Excess was removed, and light curing was performed using a LED curing unit [1200 mW/cm^2^; Elipar Freelight 2 (3M ESPE)] according to the manufacturers' recommendations. Light intensity of the light curing unit was checked prior to use (LED Radiometer; Demetron, Kerr, Orange, CA, USA).

### CLSM specimen preparation and analyses

In ten teeth of each group the adhesive systems were labeled with 1% sodium fluorescein (FNa, Sigma Aldrich, Steinheim, Germany) prior to the application inside the root canal using the microbrushes provided by the respective manufacturer. The core materials were labeled with 1% Rhodamine-Isothiocyanat (RITC; Sigma Aldrich) and applied into the root canal as well as onto the posts and the posts were inserted as described above. After 24 h storage in 100% humidity at 37°C the tip of the post was fixed into an exactly fitting hole of a slide. Subsequently, the roots were cut into 3 slices (thickness of 2 mm) perpendicular to the long axis of the tooth by using a band saw (Exakt Apparatebau, Norderstedt, Germany). The first cut started 2 mm below the cemento-enamel junction; thus, the slices represented a coronal, a middle, and an apical location of the post space preparation. The slices were fixed onto slides and polished using silicon carbide papers up to 4000 grit (Exakt Mikroschleifsystem; Exakt Apparatebau, Norderstedt, Germany). The disks were ultra-sonicated in NaCl for 10 min in order to remove the smear layer.

**Figure 1:**
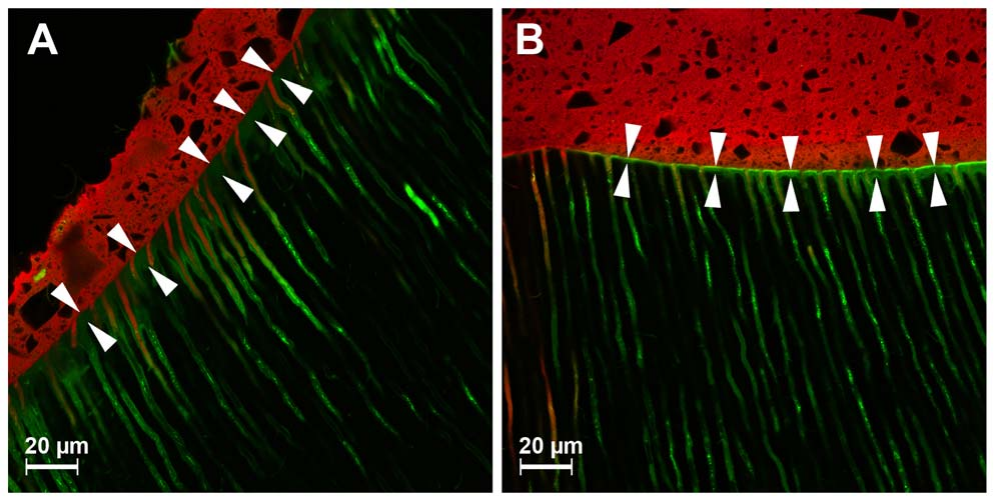
Representative microscopic images using CLSM for the measurement of the hybrid layer (indicated with arrows) for the etch-and-rinse adhesive XPBond/Self Cure Activator with Core X Flow (A) and for the self-etch adhesive system Futurabond DC with Rebilda DC (B).

**Figure 2:**
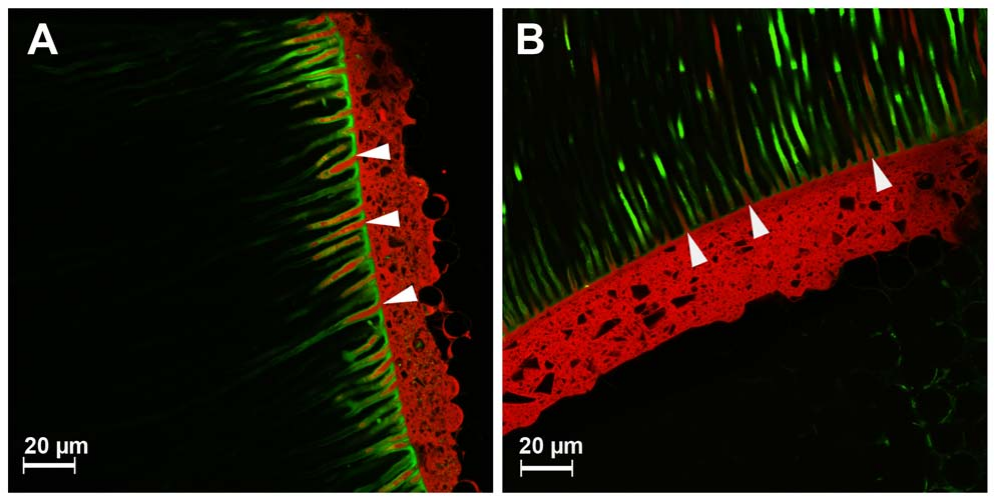
CLSM images of the etch-and-rinse adhesive system Prebond and Luxabond A&B (green) with Luxacore Z (red) (A) and the self-etch adhesive system AdheSE DC (green) used with Multicore Flow (red) (B). Arrows indicate exemplary penetration of the core material into the dentinal tubules.

CLSM analysis (Leica TCS SL; Leica, Heidelberg, Germany) was performed in dual fluorescence mode using a ×40 objective and ×2 electronic zoom. To minimize any cross talk (i. e., the simultaneous scanning of moderately overlapping emission spectra of fluorescent dyes) sequential recording of both fluorescent dyes was performed with FNa (exc: 488; em: 525/50 band pass filter) and RITC (exc. 568; em: 890 long pass filter). The size of the images was 187×187 µm^2^ with a resolution of 1024×1024 pixel. Images were recorded at 4 standardized areas of each sample, and analyzed using LEICA Software (LCS Lite Version 2611537, Leica). Recording of the topographic images of the areas started at the surface of the sample up to approximately 20 µm below the surface in 50 layers. For quantifying the thickness of the hybrid layer, measurements at 5 randomly chosen locations on each image were performed, and means were calculated (Fig. 1 A&B). The numbers of dentinal tubules penetrated with adhesive and core material were evaluated (Fig. 2 A&B).

### Push-out testing

After the microscopic analysis a 1 mm thick slice was cut off from each labeled and microscopically analyzed sample using a band saw (Exakt Apparatebau) under constant water cooling and micro push-out testing was performed (Universal testing machine Zwick; Roell, Ulm, Germany) at a cross-head speed of 0.5 mm/min. With regard to the tapered design of the post, three different sizes of punch pins, as well as three different openings, were used for the push-out testing [18].

The maximum stress was calculated from the recorded peak load divided by the computed surface. In order to calculate the exact bonding surface, the tapered design of the posts with regard to the respective part of the post was considered. Therefore, each specimen was measured using a micrometerscrew (MitutoyoMessgeräte,Neuss,Germany) and analyzed using a stereomicroscope (DV 4; Zeiss, Jena, Germany) to determine the cross-section dimension. The bonding surface was calculated using the formula of a conical frustrum 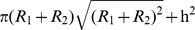 and bond strengths was evaluated accordingly as previously described [18]–[20]. The roots of the unlabeled specimens were sectioned perpendicular to the long axis of the root into six slices (thickness 1 mm) and the push-out tests were performed as above described.

### Failure mode analyses

After the push-out test each specimen was observed using a stereomicroscope (DV 4; Zeiss) at magnification ×40 to determine the failure mode. A scoring system was applied according to the failure modes: (1) adhesive failures between dentin and core material; (2) adhesive failures between post and core material; (3) mixed failures; (4) cohesive failures inside the post. The labeled specimens were additionally evaluated using the CLSM; analyses of the failure mode were performed at two previously analyzed areas in dual fluorescence mode using a ×40 objective and ×2 electronic zoom. The failure modes were classified as: (1) cohesive failure within the hybrid layer; (2) adhesive failure between hybrid and adhesive layer and core material; (3) cohesive fracture within the core material; (4) adhesive failure between post and core material. In addition, each slice was checked for the appearance of inhomogeneities (e.g. voids and bubbles) using the stereomicroscope.

### Statistical analysis

Statistical analysis was performed using IBM SPSS version 19.0 software (SPSS, Chicago, IL, USA). The alpha (Type I) error level was set to 0.05. One tooth was considered as a statistical unit; consequently data were aggregated using the break variables tooth and location. A repeated measurement ANOVA with the inter-individual factors material and incorporation of fluorescent dyes was applied at four (G RB, LC, CX, MC) and two levels (yes/no) and location as intra-individual factor (coronal, middle, apical) was applied. To examine differences between materials Tukey's post-hoc test was used. Analysis of the failure modes was conducted using crosstabs and Chi-square test.

## Results

CLSM analyses revealed significant differences between groups with respect to the factors hybrid layer thickness (p<0.0005), number of penetrated dentinal tubules with adhesive and core material (p = 0.02) and number of penetrated dentinal tubules filled with core material (p = 0.019; Repeated Measurement ANOVA). Hybrid layer thickness [mean (SD)] of G RB [1.7 (0.7) µm] and G MC [1.9 (0.6) µm] was significantly lower compared to G LC [4.8 (1.8) µm] and G CX [4.8 (0.9) µm] (p<0.0005; Tukey HSD). No effects of the location inside the root canal on hybrid layer thickness could be observed (p>0.05; Repeated Measurement ANOVA).

The factor number of dentinal tubules penetrated with adhesive and core material of G CX [14.2 (5.9)] was significantly lower compared to G MC [18 (7.4)], G LC [18.4 (7.2)] and G RB [19.5 (8.2)] (p<0.05; Tukey HSD). The factor number of penetrated dentinal tubules was significantly affected by the location inside the root canal (p<0.0005; Repeated Measurement ANOVA), a lower number of tags was observed in the apical part of the post space preparation (Table 2).

**Table 2:** Means and (standard deviations) of the factor dentinal tubules filled with adhesive and core material of the four investigated post-and-core systems.

Core Material	Number of tags cervical	Number of tags middle	Number of tags apical	Significant difference between materials (Tukey HSD)
RB	23 (4.7)	19.6 (3.1)	16 (5.8)	A
LC	20.9 (6.2)	19.6 (4.8)	14.8 (4.8)	A
CX	15.7 (2.9)	14.4 (3.9)	13 (4.3)	B
MC	19.6 (5)	18.4 (3.6)	16.1 (7.9)	A

Data with the same uppercase letter within each column indicate not significant differences (p<0.05).

Bond strength was significantly affected by the use of fluorescent dyes (p = 0.036), the core material (p = 0.001), and the location inside the root canal (p<0.0005). The interaction between core material and use of fluorescent dyes was not significant (p = 0.195; Repeated Measurement ANOVA). CX [7.7 (4.4) MPa] demonstrated significantly lower bond strength compared to LC [14.2 (8.7) MPa] and G RB [13.3 (3.7) MPa] but did not differ significantly from MC [11.5 (3.5) MPa]. Bond strengths were reduced in labeled specimens and in the apical part of the post space preparation (Table 3). No differences in bond strengths between the four investigated post-and-core systems could be detected for the unlabeled specimens (p = 0.311; Repeated Measurement ANOVA) (Table 4).

**Table 3:** Means and (standard deviations) of the factor bond strength of labeled specimens of the four investigated post-and-core systems.

Core material	Labeled Bond strength cervical MPa	Labeled Bond strength middle MPa	Labeled Bond strength apical MPa	Significant difference between materials (Tukey HSD)
RB	15.4 (5)	11.1 (4)	9.9 (5.6)	A
LC	17.8 (10.3)	15.7 (10.7)	11.6 (10.2)	A
CX	6.1 (3.7)	5.2 (2.5)	4 (2.4)	B
MC	10.2 (4.8)	9.6 (3.2)	8.4 (4.5)	AB

Data with the same uppercase letter within each column indicate not significant differences (p<0.05).

**Table 4:** Means and (standard deviations) of the factor bond strength of unlabeled specimens of the four investigated post-and-core systems.

Core material	Unlabeled Bond strength cervical MPa	Unlabeled Bond strength middle MPa	Unlabeld Bond strength apical MPa	Significant difference between materials (Tukey HSD)
RB	17.4 (3.5)	14.7 (4.8)	11.5 (4.6)	A
LC	12.7 (11.4)	12.5 (7.4)	15 (7.9)	A
CX	14.6 (8.2)	10 (5.3)	6.3 (3.4)	A
MC	20.1 (4.6)	13.4 (6)	7.4 (3.8)	A

Data with the same uppercase letter within each column indicate not significant differences (p<0.05).

The failure modes of the labeled and unlabeled specimens analyzed using the stereomicroscope are presented in Table 5 and 6 and were affected by the material (p<0.0005; Chi-square), but not by the use of the fluorescent dyes (p = 0.226; Chi-square). Failure modes analyzed using CLSM were not affected by the investigated systems (p = 0.193; Chi-square). The occurrence of voids was affected by the investigated materials for the labeled (p = 0.012) and the unlabeled specimens (p = 0.039; Chi-square), respectively (Table 6).

**Table 5:** Failure modes of the labeled (fluorescent dyes) investigated materials analyzed using stereomicroscope (A = adhesive between core material and dentin, M = mixed failure, AP = adhesive between post and core material, C = cohesive inside the post) and CLSM (C HL = cohesive inside the hybrid layer, A HL = adhesive between hybrid layer and core material, CC = cohesive inside the core material, AP = adhesive between post and core material).

	Stereomicroscope				CLSM			
Failure mode	A	M	AP	C	C HL	A HL	CC	AP
Rebilda DC	36.7	30	33.3	0	56.7	26.7	10	6.6
Luxacore Z	76.6	0	6.7	16.7	46.7	40	0	13.3
CoreXFlow	63.4	13.3	20	3.3	33.3	53.3	3.3	10
Multicore Flow	96.7	0	0	3.3	53.3	43.3	3.4	0

**Table 6:** Failure modes of the unlabeled investigated materials analyzed using stereomicroscope (A = adhesive between core material and dentin, M = mixed failure, AP = adhesive between post and core material, C = cohesive inside the post) and occurrence of voids in labeled and unlabeled specimens for the investigated materials.

	Stereomicroscope				Stereomicroscope voids	
Failure mode	A	M	AP	C		
Use of dyes	No				yes	no
Rebilda DC	26.7	33.3	33.3	6.7	26.7	33.3
Luxacore Z	70	13.3	10	6.7	20	13.3
CoreXFlow	56.7	13.3	30	0	10	13.3
Multicore Flow	70	3.3	0	26.7	10	6.7

## Discussion

The morphological characteristics of the resin-dentin interface significantly differed between the systems. As expected etch-and-rinse adhesive systems generated a thicker hybrid layer formation compared to self-etch adhesive systems in the present investigation, this has been reported previously [11], [21]. However, it has to be emphasized that hybrid layer thickness do not correlate with bonding effectiveness for self-etch adhesive systems [22]. The self-etching primer AdheSE (pH 1.7) and Futurabond DC (pH 1.4) can be classified as medium-aggressive self-etch adhesive systems [23]. Consequently, the measured hybrid layer thickness of these systems in the present study is in accordance with a previously described data [22]. Differentiation between hybrid layer and adhesive layer for self-etch adhesive systems has been performed as indicated by arrows in Fig. 1B and described previously [24]. Nevertheless, the morphological analysis of the adhesive interface using the dual fluorescence technique with the CLSM might have been affected by dye leaching of fluorochromes into the noninfiltrated dentin [25]. This phenomenon may occur if the dye is not well fixed to the component in which it has been incorporated, and recorded images might indicate dye distribution rather than the component to which the dye has been attached. Moreover, dye leaching of labeled adhesive systems can be affected by the solvents and insufficient polymerization [26]. Consequently, the risk of nonhomogeneous dye distribution inside the adhesive system as well as dye leaching cannot be completely eliminated. However, a previous study revealed comparable results concerning hybrid layer thickness and penetration into dentinal tubules for SEM and CLSM analyses [10].

The smear layer produced by motorized preparation such, as with post drills has been demonstrated to be greater in volume compared to hand filing of the root canal [27]. Irrigation after post space preparation was carried out with NaCl solution in the present study; consequently, no effective smear layer removal could be expected prior to the application of the investigated adhesive systems. In order to be consistent between the groups one irrigation protocol for all investigated systems irrespective of the manufacturers' recommendations was applied. Although two manufacturers (Ivoclar Vivadent for Adhese DC and Voco for Rebilda DC) of the investigated systems recommend NaOCl after post space preparation, NaCl was used as final rinse in order to avoid any effects of the irrigation protocol on bond strength, because the effects of NaOCl as a final rinse on bond strength have been discussed controversially [28]–[31]. Moreover remnants of sealer or gutta-percha might hamper bonding inside the root canal [32], [33]. Consequently, the cleanliness of the post space was checked using an operating microscope (OPMI pico, Zeiss, Jena, Germany) (magnification 23×), because it has been shown that additional pre-treatment of the root canal dentin using sandblasting with Al_2_O_3_ or rotating brushes do not enhance the cleanliness of the root canal as well as bond strength inside the canal [32]. It has been reported that smear layer denseness may compromise dentin bonding more than smear layer thickness, especially for self-etch adhesives [34]. The present results indicate successful smear layer modification for the investigated self-etch adhesive systems, since a thinner (compared to etch-and-rinse adhesives), but continuous hybrid layer was detected inside the root canal.

The number of penetrated dentinal tubules with adhesive and core material of both self-etching adhesives investigated in the present study was even higher compared to the etch-and-rinse system XP Bond/SCA, demonstrating a good penetrating ability of these systems in spite of the smear layer inside the root canal. However, these results are in contrast to a previous CLSM analysis that revealed no significant differences in the density of infiltrated dentinal tubules between the systems XP Bond/SCA and AdheSE DC [35]. The use of activators in the cited study increased the density and quality of resin tags for all types of the investigated adhesive systems significantly. In the present study, all adhesive systems under investigation were dual-cured adhesive systems. Fifth-generation dual-polymerizing bonding agents contain coinitiators, such as benzene sulfinic acid sodium salt [36]. The initiator-catalyst system should promote adhesion of compatible dual-curable resin-based luting agents to the adhesive layer and accelerate their polymerization [37]. Consequently, differences in light transmission ability of the investigated posts [38] should not hamper the bonding performance of the systems.

The number of dentinal tubules filled with adhesive and core material was significantly affected by the location inside the root canal, so in the apical part of the post space preparation a significantly lower number of tags was visualized. This corresponds to the morphological characteristics of the root canal with a decreasing number of dentinal tubules in the apical part of the root canal [5], [39], [40]. In the present study three slices of each root have been analyzed using CLSM. On each slice four standardized areas have been selected for the analysis resulting in 12 images per specimen that have been investigated for the analyses of the morphological factors. This procedure should have guaranteed a representative overview about the amount of penetrated tubules although it cannot be ensured that all tags have been visualized tri-dimensionally. However, each topographic image was recorded up to 20 µm below the surface in 50 layers.

The null hypothesis of the present study has to be rejected, since bond strength of fiber posts inside the root canal has been affected by the investigated post-and-core systems, the incorporation of fluorescent dyes into the adhesive systems, and the location inside the root canal.

The thin push-out test is considered as a valid method to analyze bond strengths of fiber posts to root canal dentin, because the test demonstrated a more homogenous stress distribution by Finite Element analysis compared to the microtensile bond strength and less variability in mechanical testing [41].

The present study detected a thin but continuous hybrid layer for the self-etch adhesive systems, demonstrating their demineralizing capacity. This is in accordance with the results of D'Alpino et al. who found that the incorporation of fluorescent dyes into adhesive systems did not result in a significant change of pH [42]. Nevertheless, bond strength was reduced for the labeled specimens. It has been demonstrated that fluorescent dyes absorb light, consequently, they might inhibit light transmission to the photoinitiators and reduce polymer conversion and its resulting bond strength [42]. The interaction between core material and use of fluorescent dyes was not significant. Consequently, the reduction in bond strength was found for all systems and not caused by a certain material.

Bond strength of the investigated post-and-core systems inside the root canal differed solely for the labeled specimens; the unlabeled groups did not reveal any significant differences between the systems. A previous study also showed no difference in bond strength between self-etch and etch-and-rinse adhesives inside the root canal [13], whereas others reported lower bond strength for etch-and-rinse adhesive systems compared to self-etch adhesives [11], [14]. In contrast, another study showed lower bond strength for the self-etch approach compared to the etch-and-rinse or self-adhesive approach [15]. These conflicting results imply that bond strength inside the root canal is more product-dependent than affected by the adhesive approach. Data of testing core materials for luting fiber posts varied greatly among products and adhesive systems applied [13], [43], [44]. This complicates evaluation of the present data with the existing literature. A recently published review on bond strength performance of luting cements inside the root canal demonstrated besides a high heterogeneity among studies no difference in bond strength between self-etch and etch-and rinse adhesives [45], thus confirming the present results.

The applied testing of four different post-and-core materials with all components of one manufacturer can be regarded as clinically relevant since this application will be most often used in dental practice. Moreover, the combination of bond strength testing and morphological analyses for this kind of materials has not been described in the literature up to now.

The present study investigated four different post-and-core systems. Consequently, four different types of fiber posts have been used, which might have affected the present results, since it has been demonstrated previously, that push-out bond strength was more dependent on the type of fiber post than on the type of the luting agent used [46]. However, when testing a post-and-core system of one manufacturer possible incompatibilities between the materials should be excluded and the full potential of each system under laboratory conditions can be assessed [15]. This corresponds to the observed failure modes, most failures were adhesive between dentine and core material as described previously for push-out bond strength analyses inside root canals [11], [13]–[15]. Adhesive failures between post and core material were observed to a lesser extent in the present investigation, showing a good compatibility between posts and core material.

In correspondence to the morphological analyses push-out bond strengths of all investigated systems was also significantly reduced in the apical part of the post space preparation. These results corroborated a previous review that summarized that bonding in the apical region is less predictable [47].

**Figure 3:**
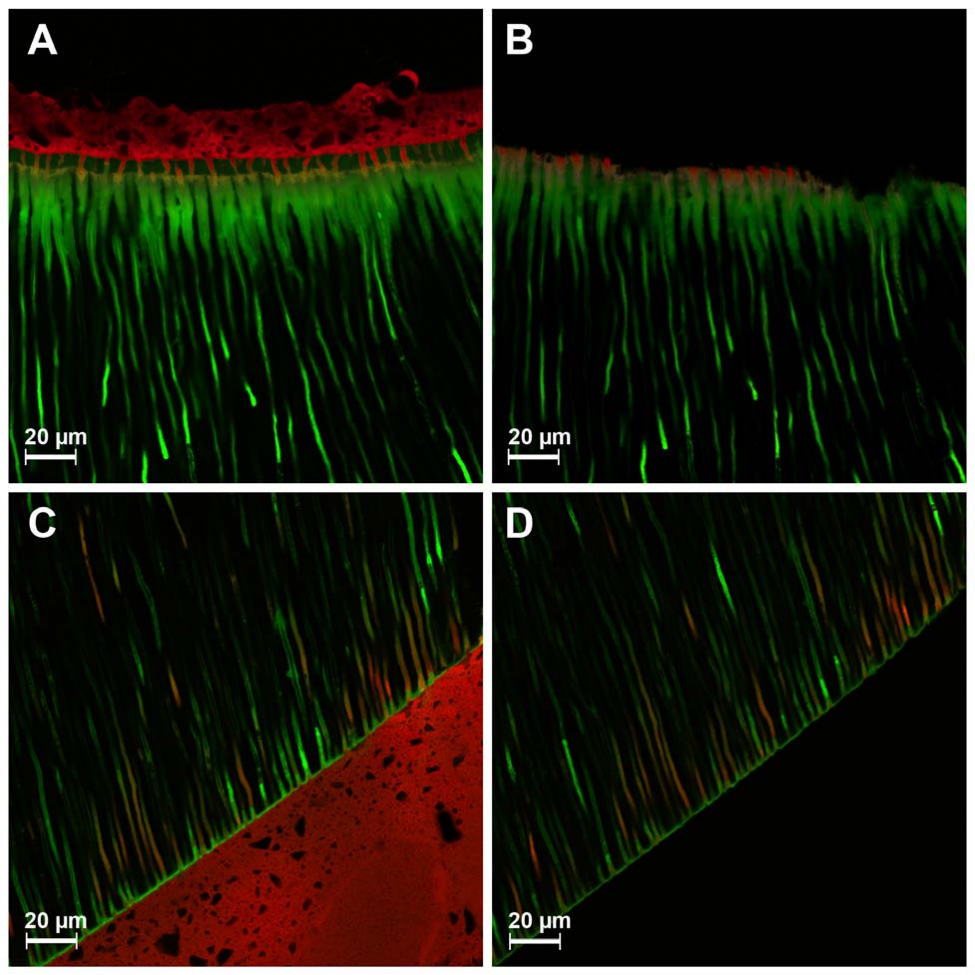
CLSM analyses of the adhesive failure mode between dentine and adhesive system. A: Prebond/Luxabond A&B/Luxacore Z before the push-out test, the application of Prebond and Luxabond A&B consecutively resulted in two different green shades inside the hybrid layer. B: Same specimen and location after the push-out test revealed a cohesive failure inside the hybrid layer and fractures of resin tags filled with core material. C: Futurabond DC/Rebilda DC before the push-out test. D: Same specimen and location after the push-out test demonstrated an adhesive failure between hybrid and adhesive layer and the resin cement.

The failure modes of the labeled specimens were analyzed after push-out testing using the stereomicroscope and subsequently using the CLSM. Adhesive failures between dentin and cement assessed using the stereomicroscope showed two different variations of failures under the CLSM: cohesive failure inside the hybrid layer (Fig. 3 A&B) and adhesive failure between hybrid and adhesive layer and resin cement (Fig. 3 C&D). These results show that the CLSM analyses of the failure modes allows a detailed description of failures as described previously [11].

All investigated core materials revealed voids in the cement layer. The present experimental set-up has limitations, since the core materials have been applied onto the posts' surfaces and into the orifices of the root canals. The application tips provided by the respective manufacturers have not been used, although beneficial effects of application aids on the occurrence of inhomogeneities inside the resin cement layer have been described [48]. The selected procedure in the present study has been attributed to the mixing process of the dyes into the core materials that did not allow the use of the provided application tips. In order to achieve a homogeneous experimental set-up, the same procedure was chosen for the unlabeled specimens. The present data did not show any correlations between the measured bond strength values and the occurrence of voids, since Rebilda DC revealed more voids compared to the other materials but no lower bond strength values. Possible beneficial effects of voids inside the cement layer have been discussed previously [33], [49]. Voids may compensate the deleterious effect of a high C-factor inside the root canal by stress relaxation provided by the air in the structure of the cement. Nevertheless, a trade-off between incorporation of air and physical properties of composite materials exists [50]. Consequently, air bubbles may also weaken the composite substantially.

It has been reported previously that one stage postendodontic restorations allowing simultaneous post cementation and core fabrication might be detrimental due to higher polymerization stress and reduced bond strength because of the increased percentage of fillers being necessary for core build-up materials [12]. However, in the present investigation the investigated post-and-core systems demonstrated comparable bond strength values irrespective of the filler content of the core materials (Table 1). Further *in vivo* and *in vitro* studies are mandatory to investigate the long-term clinical performance of these adhesives and core materials probably with an even lower filler content as well as to compare the performance between core materials and luting cements before general recommendations can be given.

## Conclusions

Bond strengths of the four investigated post-and-core systems inside the root canal were not affected by the adhesive approach. Morphological evaluation of the resin-dentine interface demonstrated homogenous hybrid layers and penetration into the dentinal tubules for all investigated systems indicating effective adhesion inside the root canal.
