# Correction: Analysis of Resin-Dentin Interface Morphology and Bond Strength Evaluation of Core Materials for One Stage Post-Endodontic Restorations

**DOI:** 10.1371/journal.pone.0118109

**Published:** 2015-02-02

**Authors:** The PLOS ONE Staff

In the “Push-out testing” subsection of the Materials and Methods, the formula of the conical frustrum is incorrect. The corrected formula, where R1 represents the coronal and R2 the apical arc radius of the post segment and h is the thickness of the slice, is: A = π(R1+R2)√(R1-R2)2 + h2.
